# Changes in Depressive Symptoms, Stress and Social Support in Mexican Women during the COVID-19 Pandemic

**DOI:** 10.3390/ijerph18168775

**Published:** 2021-08-19

**Authors:** Nadya Y. Rivera Rivera, Laura McGuinn, Erika Osorio-Valencia, Sandra Martinez-Medina, Lourdes Schnaas, Rosalind J. Wright, Martha Maria Téllez-Rojo, Robert O. Wright, Marcela Tamayo-Ortiz, Maria José Rosa

**Affiliations:** 1 Department of Environmental Medicine and Public Health, Icahn School of Medicine at Mount Sinai, New York, NY 10029, USA; nadya.riverarivera@mssm.edu (N.Y.R.R.); laura.mcguinn@mssm.edu (L.M.); rosalind.wright@mssm.edu (R.J.W.); robert.wright@mssm.edu (R.O.W.); 2 National Institute of Perinatology, Mexico City 11000, Mexico; erikaosorio4@hotmail.com (E.O.-V.); sandys65.sm@gmail.com (S.M.-M.); lschnaas@hotmail.com (L.S.); 3 Department of Pediatrics, Kravis Children’s Hospital, Icahn School of Medicine at Mount Sinai, New York, NY 10029, USA; 4 Center for Nutrition and Health Research, National Institute of Public Health, Cuernavaca 62100, Mexico; mmtellez@insp.mx; 5 Occupational Health Research Unit, Mexican Institute of Social Security (IMSS), Mexico City 06600, Mexico; marcela.tamayo@imss.gob.mx

**Keywords:** COVID-19, stress, depression, social support, women

## Abstract

The aim of this study was to examine changes in depression, stress and social support levels before and during the COVID-19 pandemic in women living in Mexico City. We studied 466 women enrolled in the Programming Research in Obesity, Growth, Environment and Social Stressors (PROGRESS) study who completed the Edinburgh Depression Scale (EDS) questionnaire prior (2018–2019) and during the lockdown period of the pandemic (May–November 2020). Psychosocial stress and social support for both time periods were ascertained using the Crisis in Family Systems (CRISYS) questionnaire and the Social Support Network (SSN) Scale, respectively. Associations between stress, social support and change in EDS score/depression were analyzed using generalized linear models adjusting for covariates. Higher stress (>median) during the pandemic was associated with an increase in EDS score (β: 2.13; 95% CI (1.06, 3.19), *p* < 0.001), and higher odds of depression (OR: 3.75; 95% CI (2.17, 6.50), *p* < 0.001), while social support was associated with lower odds of depression (OR: 0.56, 95% CI (0.32, 0.97), *p* = 0.037). Higher levels of stress during the pandemic were associated with depression. Social support may act as a buffer for the effects of psychosocial stress. Future studies should examine the long-term effects of stress associated with the pandemic on mental and overall health.

## 1. Introduction

According to the Centers for Disease Control (CDC), the Coronavirus Disease 2019 (COVID-19) pandemic has been linked to adverse mental health outcomes related to the morbidity and mortality caused by the disease and to mitigation activities such as stay-at-home orders and physical distancing [1]. People’s daily life and economic well-being were severely impacted by pandemic prevention measures, such as mandatory school closures and the suspension of all nonessential commercial activities. Regardless of whether the preventive measures succeeded in controlling the outbreak, the widespread lockdown can have significant psychological effects [2]. While the impact of COVID-19 on physical health is now better understood, elucidating the burden of a disease outbreak on mental health is also fundamental [3]. 

Several studies, mostly from the United States and Europe, have reported on the longitudinal impact of the COVID-19 pandemic on mental health. In late June 2020, about 4 months into the pandemic, 31% of adults in the United States reported struggling with anxiety and/or depression symptoms [1]. A study conducted in the UK found that women, participants from more socially disadvantaged backgrounds, and participants with pre-existing mental health problems had worse mental health outcomes during the pandemic [4]. Another study conducted in the UK found that population prevalence of mental distress rose from 18.9% in 2018–2019 to 27.3% in April 2020, one month into lockdown [5]. A different study from the UK found that anxiety and depression during the pandemic were greater in younger participants, women, those with pre-existing mental/physical health conditions and individuals experiencing socioeconomic adversity, even when controlling for pre-pandemic anxiety and depression [6]. Low–middle income (LMIC) countries may be particularly vulnerable to the stress caused by the eminent risk of infection and economic uncertainty [3]. LMICs usually have large populations living in overcrowded conditions where it might be impossible to physically distance. Clean water may not be readily available in every household, and supplies such as hand sanitizer are very difficult to find [7]. Furthermore, accomplishing the stay-at-home recommendations can be impossible for some families due to the economic burden.

Sanitary Emergency Measures were implemented in Mexico in March 2020, requiring the suspension of nonessential activities as well as all educational activities [8]. A study conducted in Mexico reported that half of the respondents rated the psychological distress of the outbreak as moderate to severe, 19.8% reported moderate to severe stress levels and 15.7% of respondents reported moderate to severe depressive symptoms [9]. While there is cross-sectional evidence of the impact of the pandemic on mental health in Mexico and other LMIC countries, few studies have examined longitudinal changes in mental health outcomes prior to and during the pandemic. Women, particularly those who are pregnant or postpartum, might be particularly vulnerable to the stress and anxiety experienced during the pandemic and may be at higher risk of developing mental health problems [10,11,12,13]. Moreover, school closures associated with health emergencies increase parental stress, particularly for mothers, who typically do the largest share of childcare and eldercare in most parts of the world [10]. A multi-national study found higher levels of stress and mental health difficulties among female caregivers when compared to male caregivers, suggesting that pandemic-related disruptions led to a disproportionate burden of caregiving activities [14]. Public health recommendations for physical distancing and stay-at-home orders also have limited the access to different support systems for children and families [15]. Social support (high-quality supportive relationships) has been shown to be protective of both physical and mental health and an important moderator of stress in the context of mass disasters and pandemics [16,17]. Social support provided by spouses, family members, friends and neighbors has been linked to lower biological stress responses, such as reduced cortisol levels, enhanced resiliency to stress and more effective coping with stressful situations [17]. However, the impact of social support during the pandemic on longitudinal mental health outcomes has not been elucidated. 

We leveraged longitudinal data on stress, social support and depression from the Programming Research in Obesity, Growth, Environment and Social Stressors (PROGRESS) cohort study in Mexico City to begin to fill a number of these research gaps. Specifically, we examined changes in depressive symptoms, psychosocial stress and social support prior (2018–2019) and during the pandemic (2020) in women enrolled in this cohort. We also examined the association between psychosocial stress and social support during the pandemic as a predictor of the change in depressive symptoms. 

## 2. Materials and Methods

### 2.1. Study Population

The PROGRESS study recruited pregnant women in primary care clinics of the Mexican Social Security Institute in Mexico City between July 2007 and February 2011 [18]. Women who met the following inclusion criteria were recruited: <20 weeks gestation, singleton pregnancy, at least 18 years of age, had completed primary education, planned to stay in Mexico City for the next 3 years, had access to a telephone, had no medical history of heart or kidney disease, did not consume alcohol daily, no drug addiction and did not use any steroid or anti-epilepsy medications [18]. Procedures were approved by institutional review boards at the Harvard School of Public Health, Icahn School of Medicine at Mount Sinai, and the Mexican National Institute of Public Health. Women provided written informed consent. The data included in these analyses were collected at two time points, during an in-person study visit prior to the pandemic (2018–2019), which corresponds to the index child’s age 8 study visit and via a telephone call during the pandemic (May–November 2020). In this study, 466 women had complete data at both time points for analyses. 

### 2.2. Psychosocial Stress: Negative Life Events

Psychosocial stress was measured using the Crisis in Family Systems-Revised (CRISYS-R) survey, validated in Spanish [19]. Previous work has shown the CRISYS to be reliable in both English and Spanish-speaking populations [19,20,21]. In this survey, women were asked to endorse life events experienced in the past six months across 11 domains: financial, legal, career, relationships, safety in the home, safety in the community, medical issues pertaining to self, medical issues pertaining to others, home issues, authority and prejudice, and to rate each as positive, negative or neutral. Research suggests there is increased vulnerability when experiencing events across multiple domains, as this circumstance is more likely to overwhelm coping resources; therefore, the number of domains with one or more events endorsed as negative were summed to create a negative life events domain score (range 0–11), with higher scores indicating greater stress, as done in prior research [22,23,24]. We also examined the report of negative life events (NLEs) in each individual domain by creating a dichotomous variable for whether or not there was a report of a negative life event in each domain. Details on the domain-specific questions can be found in the Supplemental Table S1. 

### 2.3. Depression Symptoms 

Women completed the validated Spanish version [25,26] of the Edinburgh Depression Scale questionnaire (EDS) interview prior to and during the COVID-19 pandemic. The 10-item EDS asks about depression symptoms in the past 7 days, including: “1: I have laughed and been able to see the funny side of things,” “2: I have looked forward with enjoyment to things,” “3: I have blamed myself unnecessarily when things went wrong,” “4: I have been anxious or worried for no good reason,” “5: I have felt scared or panicky for no very good reason,” “6: Things have been getting on top of me,” “7: I have been so unhappy that I have had difficulty sleeping,” “8: I have felt sad or miserable,” “9: I have been so unhappy that I have been crying,” and “10: The thought of harming myself has occurred to me.” Participants rated the severity or frequency of each item based on 4 levels scored from 0 (indicating the most favorable condition) to 3 (indicating the least favorable condition) for each item. Total scores can range from 0 to 30. The Cronbach alpha was 0.74 for the pre-pandemic EDS and 0.73 for the pandemic EDS. 

### 2.4. Social Support Network 

The Spanish version of the Social Support Network (SSN) Scale was used to assess participants’ social support networks both prior and during the pandemic. This instrument has been previously validated in Mexican populations [27] and showed internal consistency and construct validity [28]. Participants rated their degree of agreement with each item based on 4 levels, scored from 1 (indicating strongly disagree with this statement) to 4 (indicating strongly agree with this statement) for each item. Scores ranged from 4 to 20, with higher scores indicating greater social support. The Cronbach alpha was 0.89 for both the pre-pandemic and pandemic SSN scale.

### 2.5. Covariates

Covariates were selected a priori and included women’s pre-pandemic socioeconomic status (SES) and age (continuous in years) at the time of the pandemic. Socioeconomic status (SES) was calculated based on an index created by the Mexican Association of Market and Public opinion Research Agencies (Spanish acronym AMAI) using 13 variables derived from questionnaire results [29]. This measurement has been validated in the National Survey on Health and Nutrition (ENSANUT) in Mexico, the equivalent of the National Health and Nutrition Examination Survey (NHANES). These levels were then collapsed into lower, medium, and higher SES. In sensitivity analyses, the month of the pandemic phone call was also included as a covariate. For a more balanced distribution, months with a low number of calls were collapsed (May–June, July, August, September–November). 

### 2.6. Statistical Analyses

NLE domain scores for both time points were dichotomized at the median, defined as low stress (NLE score ≤ 3) and high stress (NLE score > 3). Social Support Network Scores were also dichotomized at the median (score > 17) for both time points. Change in depressive symptoms was calculated by subtracting the baseline total EDS score from the EDS score during the pandemic. Depression was also examined as a dichotomous outcome using EDS scores at a clinically-relevant cutoff (EDS score > 12) [30]. We performed descriptive statistics for dependent and explanatory variables. Generalized linear models were used to examine the association between stress, social support and change in depressive symptoms. Final models included NLE score and social support score at baseline, NLE score and social support score during the pandemic, SES at baseline and woman’s age during the pandemic call. Models estimating the odds of depression using the relevant cutoff were additionally adjusted for depression at baseline. 

## 3. Results

Table 1 shows the distribution of women’s characteristics before and during the pandemic. We did not find any significant differences in the distribution of EDS scores or the proportion of participants with probable depression (EDS score > 12). There was a reduction in NLE scores during the pandemic when compared to baseline. Social support was also higher prior to the pandemic. A smaller proportion of participants reported negative life events in the authority, career, home, safety in the home and legal domains. There was a marginal increase in negative life events in the financial domain. We did not find any significant differences in participant characteristics when comparing the women who did not have data for the pandemic period compared to those included in our sample (Table S2). 

**Figure 1:**
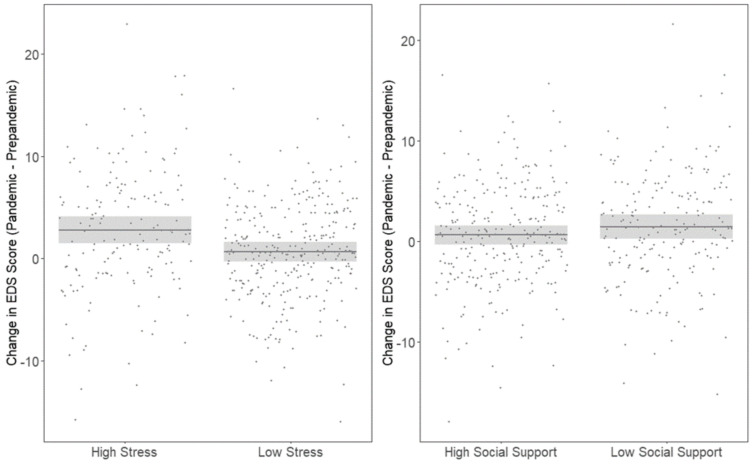
Association between change in EDS score, stress and social support during the pandemic. Model adjusted for NLE and social support score at baseline and during the pandemic, SES at baseline and age during pandemic.

**Figure 2:**
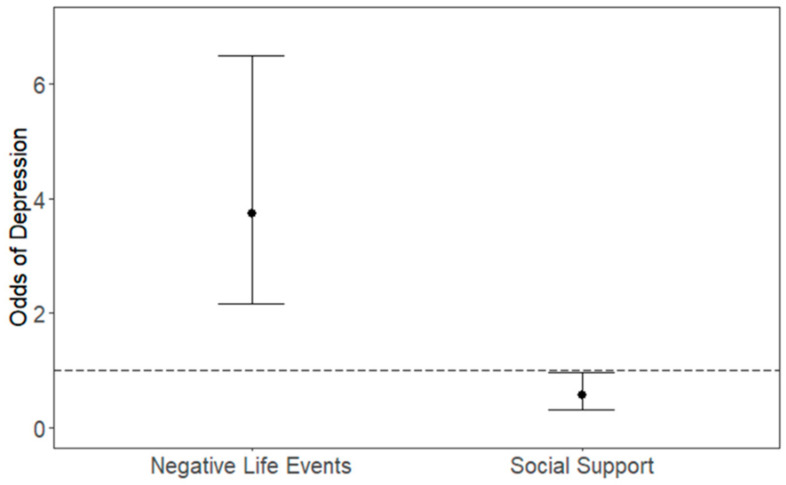
Association between odds of depression, stress and social support during the pandemic. Model adjusted for NLE and social support score at baseline and during the pandemic, SES and depression at baseline and age during pandemic.

Higher stress (> median) during the pandemic was associated with a greater change in EDS score (β: 2.13; 95% CI (1.06, 3.19), *p* < 0.001) while social support was associated with a decrease in EDS score, albeit this association did not reach statistical significance (β: −0.82; 95% CI (−1.83, 0.20), *p* = 0.115) as shown in Figure 1. We also found that higher stress during the pandemic was associated with higher odds of depression (OR: 3.75; 95% CI (2.17, 6.50), *p* < 0.001) while social support was associated with lower odds of depression (OR: 0.56, 95% CI (0.32, 0.97), *p* = 0.037) as shown in Figure 2. Higher stress during the pandemic in particular domains, including personal relationships (β: 1.84 (95% CI: 0.87, 2.81) home (β: 1.44; (95% CI 0.26, 2.62)) and financial domains (β: 1.84; (95% CI: 0.85, 2.83)) were associated with an increase in EDS score as shown in Table 2.

We performed sensitivity analyses that included adjustment for the month of EDS assessment during the pandemic, and we did not see any changes in the association between our main predictors (stress and social support) and change in EDS score and odds of depression (see Supplemental Table S3).

## 4. Discussion

We found that experiencing higher stress during the pandemic was associated with an increase in depression symptoms and higher odds of depression in women living in Mexico City. Experiencing negative life events in particular domains, including personal relationships, home and financial were associated with an increase in depression symptoms during the pandemic. Social support during the pandemic had a protective effect and was associated with lower odds of depression. Given that the entirety of the Mexico City population lived under the social restriction created by the pandemic, the results are best interpreted as modifiers of the impact of the pandemic rather than direct effects. Such widespread changes in the social and physical environment are rare, and situations such as these represent natural experiments, which can be extremely useful, particularly because there may be future pandemics, and learning from the experience of COVID-19 is paramount and will assist in planning mental health priorities and resources in future large-scale events. 

Previous studies had reported worse mental health outcomes during the pandemic [4], increases in mental distress [5] and anxiety and depression [6,31], mostly in high-income countries. Unlike other reports from LMIC, which were largely cross-sectional or time series [9,32], we conducted a longitudinal analysis of depressive symptoms (i.e., within the identical group of subjects before and during the pandemic). This allowed us to address the change in depression over time, unlike the other reports. While we did not find significant differences in the prevalence of depression before and during the pandemic, we did find that the direction of the change in depressive symptoms during a global pandemic was predicted by both negative life events and social support. A study conducted in Mexico City found a sharp increase in calls reporting anxiety during the pandemic to a 24-h government-funded call center [33]. Despite the rise in inquiries for anxiety, this study found no impact on depression [33]. Another study in Brazil did not find evidence of increases in rates of common mental disorders and depression when comparing two pre-pandemic assessments (2008–2010 and 2016–2018) and the initial phases of the COVID-19 pandemic (May–July 2020) [34]. An important aspect of the longitudinal nature of our study is that we could assess the change at the individual level. While the population levels of depression did not change, suggesting that the pandemic had an equal impact across all participants as a whole, we were nonetheless able to see differences in depressive symptoms when taking into account stressful events and social support. The response, therefore, was contextual and required measures of baseline stress and social support to understand it. Another factor to take into consideration is the concept of “familism”, whichrefers to tight social and familial structures that include strong family identification, attachment, mutual support, family obligation and familial interconnectedness common in Latino cultures [35]. Familism could explain why we did not see a change in the prevalence of depression in Mexico as was reported in other countries, such as the UK, Canada and the US.

We expected there would be an increase in perceived levels of stress during the pandemic because of the quarantine and preventive measures, as well as financial uncertainty. Parents reported feelings of anxiety, fear and depression because of limited financial and social resources, unemployment and reported increased use of alcohol and other substances [10]. A study conducted in Italy found that women were particularly vulnerable; any recent COVID-19-related stressful life event was associated with higher levels of post-traumatic stress symptoms, depression, anxiety, insomnia, perceived stress and adjustment disorder during the pandemic [36]. In this same study, discontinued working activity due to the COVID-19 pandemic was associated with all the outcomes [36]. While we did not find any changes in overall stress levels when comparing the pre-pandemic and pandemic periods, we found slight increases in the proportion of women reporting negative life events in the personal relationships and financial domains, two areas particularly impacted by pandemic events and mitigation activities. 

Furthermore, we also found that higher stress in those particular domains was associated with an increase in depression symptoms during the pandemic. Studies in the U.S. have shown that factors including lower-income, having less than USD 5000 in savings, and having exposure to more stressors were associated with a greater risk of depression symptoms during the COVID-19 pandemic [31]. Another study conducted in Canada found that women who had income disruptions, difficulty balancing homeschooling with work responsibilities and difficulty obtaining childcare had larger increases in depression and anxiety symptoms [37]. Relationships and home environments were reportedly associated with depression in other studies as well. A study conducted in Peru found that that 8.3% of participants experienced an increase in physical violence within their households during the lockdown period [38]. The proportion of women experiencing domestic violence was also three times higher for those who had already reported it in a previous assessment in 2016, with 23.6% reporting an increase during this time [38]. 

We found evidence of higher social support and lower odds of depression. Social support (ties to family, friends, community and social groups) has repeatedly been shown to buffer the association between psychosocial stress and both physical and psychological morbidity [39]. Social isolation, in general, has been linked to an array of adverse health outcomes. Greater social network diversity has been related to less anxiety, depression and nonspecific psychological distress. Social support may reduce or buffer the deleterious effects of stress by altering the perception of a situation [40]. Both animal and human studies have shown that social support reduces stress-induced biological responses, such as cortisol release [41,42,43], and social support may reduce negative appraisal.

Our study had many notable strengths. The PROGRESS cohort is an established prospective birth cohort with well-characterized demographic and covariate data. We were able to examine longitudinal changes in depression symptoms, psychosocial stress and social support at the individual level both prior to and during the pandemic. PROGRESS is an urban population, and our results may translate to other populations who face similar stressors. Similar studies conducted in other regions may help elucidate whether these results generalize to other populations. We also acknowledge some limitations. While we adjusted for several other variables that may confound the association between stress and depression, we cannot rule out the presence of residual confounders that may explain our associations. For example, previous history of mental illness [44], alcohol or drug use [45] and experiencing interpersonal violence [46] may impact the association between stress and depression. Our sample consisted only of women with school-aged children. While the original study excluded daily alcohol drinkers, we did not consider current alcohol consumption in our analysis. The EDS questionnaire was applied in person during the baseline period, while during the pandemic, it was applied by phone. We are comparing two measurements that were applied with different methodologies, and we do not have data to isolate the effect of the change in methodology. The CRISYS-R questionnaire was also validated in native Spanish speakers in the United States, not in Mexico. We were not able to assess the direct impact of COVID-19 infections on depression, as we did not collect information on whether our participants, family members or close relatives contracted the infection. We also did not collect any information on the women’s partner’s/spouse’s mental health. We also only managed to capture a specific window of time during the pandemic (May–November 2020), although the confinement measures were similar in our study period. The Mexican government used a traffic light system, in which red meant that citizens should not go out unless it is absolutely necessary, orange meant that if they could, they should stay home, yellow meant that activities could be done with precaution and green meant that people could go out with precaution measures [47]. During the study period, Mexico City remained between the red and orange stages and schools were closed [47].

## 5. Conclusions

Our findings suggest that higher levels of stress during the pandemic lockdown were associated with increases in EDS scores and odds of depression. We also found that social support may act as a buffer that alleviates the potentially harmful impacts of stress on mental health. Potential interventions could include government organizations and public health officials promoting public awareness of mental health issues. It may be important to target specific interventions to protect women’s mental health, given they are more likely to experience a range of risk factors linked to poor mental health outcomes, such as the higher risk of mental health problems during pregnancy and the perinatal period, intimate partner violence, parenting and caregiving stress and shoulder the bulk of domestic responsibilities [10,13]. Outreach efforts via social media/internet to promote social support and mobile health-based interventions to access mental health care providers could be cost-effective and easily accessible interventions when face-to-face contact might be limited. Given the burden of financial stress due to the pandemic, economic interventions may also be beneficial. Future studies in our cohort will take into consideration how the type and length of lockdown preventive measures taken impacted mental well-being. We will also collect information on any COVID-19 cases that might have happened in the women’s household and their long-term impact.

## Figures and Tables

**Table 1:** Characteristics of women in the PROGRESS study pre-pandemic and during the pandemic.

Characteristic	Pre-Pandemic Mean (SD) or *N* (%)	Pandemic Mean (SD) or *N* (%)	*p*-Value
Participant’s age (years)		39.22 (5.54)	
SES			
Lower	174 (37.3)		
Medium	229 (49.1)		
Higher	63 (13.5)		
NLE score	3.22 (2.07)	2.92 (1.86)	0.004
Social support score	17.45 (2.57)	17.20 (2.36)	0.012
EDS score	7.48 (5.80)	7.34 (5.83)	0.630
Depression (EDS score > 12)	92 (19.5)	90 (19.1)	1.000
Proportion of at least one NLE per CRYSIS domain			
Authority	43 (9.2)	21 (4.5)	0.005
Career	75 (16.1)	66 (14.2)	0.444
Financial	223 (47.9)	244 (52.4)	0.150
Home	170 (36.5)	105 (22.5)	0.000
Safety in the home	174 (37.3)	107 (23.0)	0.000
Legal	25 (5.4)	11 (2.4)	0.022
Medical issues pertaining to self	84 (18.0)	77 (16.5)	0.556
Medical issues pertaining to others	157 (33.7)	148 (31.8)	0.550
Neighborhood safety	273 (58.6)	272 (58.4)	1.000
Relationships	212 (45.5)	241 (51.7)	0.060
Prejudice	61 (13.1)	68 (14.6)	0.520

Abbreviations: CRISYS, Crisis in Family Systems Revised; EDS, Edinburgh Depression Scale; NLE, negative life events; SES, socioeconomic status. Differences were tested using paired *t*-tests (continuous variables) and McNemar’s test (categorical variables).

**Table 2:** Associations between reporting of any negative life event in individual domains during the pandemic and change in EDS score.

Individual CRISYS Domain	Change in EDS Score β (95% CI)
Authority	1.14 (−1.26, 3.53)
Career	0.79 (−0.65, 2.24)
Financial	1.84 (0.85, 2.83) ^†^
Home	1.44 (0.26, 2.62) *
Safety in the home	1.14 (−0.06, 2.34)
Legal	−0.53 (−3.80, 2.75)
Medical issues pertaining to self	1.23 (−0.13, 2.60)
Medical issues pertaining to others	−0.05 (−1.31, 0.79)
Neighborhood safety	0.54 (−0.48, 1.56)
Personal Relationships	1.84 (0.87, 2.81) ^†^
Prejudice	1.18 (−0.25, 2.61)

Models adjusted for age, SES, individual domain score at baseline and social support. *p*-values * <0.05, ^†^ <0.001. Abbreviations: CRISYS, Crisis in Family Systems Revised; EDS, Edinburgh Depression Scale; CI, Confidence Interval.

## Data Availability

Minimal dataset may be available upon on request due to privacy restrictions.

## Supplementary Materials

The following are available online at https://www.mdpi.com/article/10.3390/ijerph18168775/s1, Table S1: CRYISIS questions by domain, Table S2: Comparison of included versus not included participant characteristics, Table S3: Association between depression, stress and social support during the pandemic adjusting for month of call during the pandemic.
