# Supplementary material for: Changes in Depressive Symptoms, Stress and Social Support in Mexican Women during the COVID-19 Pandemic
Source: Int J Environ Res Public Health. 2021 Aug 19;18(16):8775. doi: 10.3390/ijerph18168775 (PMC8394154; doi:10.3390/ijerph18168775)
Supplement: Supplementary file 1 [file ijerph-18-08775-s001.zip › ijerph-1321865-supplementary.pdf]

Supplemental Material

Table S1. CRYISIS questions by Domain

Domain	Questions
Financial	•Did your income increase by a lot?<br>•Did you go deeply in debt?<br>•Did your income decrease by a lot?<br>•Did you give money to support family or friends not living with you?<br>•Did you go without food because you didn't have the money to pay for it?<br>•Did you go without some clothing because you couldn't pay for it?<br>•Did you miss a rent or mortgage payment because you couldn't pay for it?<br>•Did the utility or phone company threaten to cut off your service because you couldn't pay the bills?<br>•Was your telephone, electricity, or gas turned off?<br>•Did you go without furniture because you did not have the money to pay for it?<br>•Did you miss an appointment or have to change your plans because you had no transportation to get there?
Legal	•Did you have legal problems? Did you go without legal advice when you needed it? Was anyone in your family pulled over or questioned by the police? Were you or your partner questioned about your legal status?

	•Did anyone in your family get arrested?<br>•Did anyone in your family go to jail? Did anyone bully your child or children? Did your child or children challenge your family values and beliefs? Did any of your children get bad grades or bad marks in school?
Career	•Did you return to school?<br>•Did you begin a new job or get promoted?<br>•Did you get laid off?<br>•Did you look for a job?
Relationships	•Did your regular child care arrangements change in any way?<br>•Did you get married? Did you and your partner disagree about raising your children? Did you and your partner disagree about your roles and responsibilities? Did you miss an important family event that you wanted to attend?<br>•Did you get a divorce or break up with a partner?<br>•Did you get back together with a partner?<br>•Did a family member die?<br>•Did a friend die?
Medical issues pertaining self	•Did you or your partner get pregnant?<br>•Did you or your partner have a baby? Did any of your children get pregnant or get someone else pregnant?<br>•Did you or your partner have a miscarriage?<br>•Did you or your partner have an abortion?<br>•Did you become ill or did you have a flare up of a chronic illness?

	•Did you get admitted to the hospital?
Medical issues pertaining others	•Did your child or children become ill or have a flare up of a chronic illness? Did you go without medical care when you needed it?<br>•Did your child or children get admitted to the hospital?<br>•Did another family member become ill?<br>•Did a friend become ill?
Home Safety	•Did you feel emotionally or physically abused?<br>•Did your child or children feel emotionally or physically abused?<br>•Were you a victim of a crime while you were in your own home?
Authority	•Did you have trouble with your teacher(s)?<br>•Did you have trouble with social service agencies?<br>•Did you have trouble with medical or health professionals?
Neighborhood safety	•Did anything happen in your neighborhood that made you feel unsafe?<br>•Were you a victim of a crime while you were outside or away from your home?<br>•Did you hear violence outside your home (for example, gunfire)?<br>•Did you see violence?<br>•Did your child or children see violence?<br>•Was your child (or were your children) a victim of a crime?<br>•Was anyone else in your household a victim of a crime?<br>•Did you see drug dealing in your building or neighborhood?
Home	•Did you lose your housing?

	•Did your child or children get into trouble? Were any of your children involved with someone who you think is a gang member?<br>•Did a relative or friend move into your home?<br>•Did a relative or friend move out of your home?<br>•Did you move?<br>•Did rats, mice, or insects bother you in your home?<br>•Did you have trouble with your landlord?<br>•Did you have trouble with your neighbors?
Prejudice	•Did you have trouble reading or understanding something that was important to you? Did you have trouble communicating with someone about something that was important to you?<br>•Did someone treat you unfairly because of your age?<br>•Did someone treat you unfairly because of your sex?<br>•Did someone treat you unfairly because of your race?<br>•Did someone treat you unfairly because you didn't have a lot of money?<br>•Did someone treat you unfairly because of the way you speak?

Table S2. Comparison of included versus not included participant characteristics

Characteristic	Included N=468 Mean (SD) or N (%)	Not included N=51 Mean (SD) or N (%)	p-value
SES			
Lower	174 (37.3)	21 (41.2)	0.288
Medium	229 (49.1)	27 (52.9)	
Higher	63 (13.5)	3 (5.9)	
Social support score	17.45 (2.57)	17.24 (2.83)	0.495
EDS score	7.48 (5.80)	7.45 (6.72)	0.975
Depression (EDS score>12)	92 (19.5)	11 (21.6)	0.729
NLE score	3.22 (2.07)	3.24 (2.09)	0.968

Abbreviations: EDS, Edinburgh Depression Scale; NLE, negative life events; SES, socioeconomic status. Differences tested using Chi-square (categorical variables) and t-test (continuous variables).

Table S3. Association between depression, stress and social support during the pandemic adjusting for month of call during the pandemic

Characteristic	Change in depression symptoms β (95%CI)	Odds of depression (95%CI)
High stress (NLE score>3)	2.22 (1.16, 3.27) ‡	3.95 (2.27, 6.87) ‡
Social Support	-0.87 (-1.88, 0.13)	3.95 (2.27, 6.87) ‡

Model adjusted for NLE and social support score at baseline, SES at baseline and age during pandemic, and month of call.

Statistically Significant by *<0.05, †<0.01, ‡<0.001
